# Spiritual Beliefs and Divine Motivation Among Disaster‐Volunteering Nurses: A Qualitative Study

**DOI:** 10.1002/nop2.70706

**Published:** 2026-07-22

**Authors:** Zahra Talebi, Abbas Ebadi, Gholamreza Mahmoodi‐Shan, Naser Behnampour

**Affiliations:** ^1^ Department of Medical Surgical Nursing, Faculty of Nursing and Midwifery Golestan University of Medical Sciences Grogan Iran; ^2^ Nursing Care Research Center, Clinical Sciences Institute Baqiyatallah University of Medical Sciences Tehran Iran; ^3^ Nursing Research Center, Faculty of Nursing and Midwifery Golestan University of Medical Sciences Gorgan Iran; ^4^ Department of Biostatistics and Epidemiology, Faculty of Health Golestan University of Medical Sciences Gorgan Iran

**Keywords:** disaster volunteering, divine motivation, Iran, nurses, qualitative research, spirituality

## Abstract

**Aim:**

To explore how spirituality and divine motivation inspire nurses to volunteer and shape their actions in challenging situations.

**Design:**

A qualitative study using a conventional content analysis was conducted.

**Methods:**

In‐depth interviews were conducted between September 2024 and March 2025 with 19 nurses selected through purposive sampling with maximum variation. All participants had engaged in disaster volunteering on at least one occasion. Data were analysed using the approach proposed by Lindgren, Lundman and Graneheim to identify key themes reflecting nurses' spiritual experiences during voluntary disaster response.

**Results:**

Three overarching themes emerged from the nurses' experiences: a spirituality‐oriented worldview, spirituality and sacredness in voluntary nursing and spirituality in the volunteering process. Nurses perceived disaster volunteering as a spiritually meaningful act rooted in faith and moral obligation, transforming it from a purely professional responsibility into an expression of worship and personal growth.

**Conclusion:**

The findings suggest that spirituality may play an important role in motivating and sustaining disaster volunteering among nurses in this context. Incorporating attention to spiritual and culturally sensitive dimensions in disaster nursing may support nurses' resilience, engagement and quality of care during disaster response.

**Implications for Nursing Practice:**

Disaster nursing practice should recognise spirituality as a potential source of motivation, resilience and psychological support for nurses. Disaster preparedness programs may incorporate spiritual coping, ethical reflection, resilience‐building and stress‐management strategies alongside practical response skills. Nursing managers can support nurses by providing access to psychological and spiritual support services, including counselling, peer debriefing and referral options when needed. Culturally responsive approaches should be integrated into care to respect diverse beliefs among nurses and affected populations. Recruitment and retention strategies should acknowledge multiple sources of motivation, including professional, altruistic, and, for some nurses, spiritual factors, to better support sustained engagement in disaster volunteering.

**Patient or Public Contribution:**

Patients and members of the public were not involved in the design or conduct of this research.

## 1 Introduction

Disasters, whether natural or human‐made, remain among the most pressing challenges of the 21st century (Centre for Research on the Epidemiology of Disasters 2025). In such contexts, volunteer nurses play a crucial role in providing multidimensional care that extends beyond clinical tasks to address the psychological, emotional and spiritual needs of affected populations (Fletcher et al. 2022; Mercan Annak et al. 2024; International Council of Nurses 2019).

Due to the complex, dynamic and unpredictable nature of disasters, volunteer nurses frequently encounter a range of challenges. These include unstable working conditions, psychological and emotional strain, limited communication and coordination infrastructure, ethical dilemmas and insufficient disaster preparedness (Farokhzadian et al. 2024; Jiménez‐Herrera et al. 2020; Akbari et al. 2024). Numerous studies have shown that many nurses, particularly during disasters, rely on inner resources, especially spiritual beliefs, to cope with intense physical and psychological pressures (Husna et al. 2021; Fincham and May 2021; Taylor et al. 2023). In such situations, spirituality and religious faith may enhance psychological resilience and facilitate meaning‐making and social reconstruction (Aten et al. 2014).

For Muslim healthcare professionals, faith may play dual roles (Amin 2025). Some interpretations may intensify distress through perceived divine displeasure or injustice, whereas core practices such as prayer, supplication and reliance on God (*tawakkul*) often function as sources of support, promoting resilience, psychological flexibility and adaptive meaning‐making.

Research has shown that prayer among Muslim nurses can reduce occupational stress and improve life satisfaction (Achour et al. 2019). Similarly, prayer and remembrance practices during the COVID‐19 pandemic were associated with a lower likelihood of post‐traumatic stress disorder symptoms (Szałachowski and Tuszyńska‐Bogucka 2021).

These findings are consistent with the concept of positive spiritual coping, which encompasses personal, social, environmental and religious dimensions (Charzyńska 2015) and underscore the role of spiritual beliefs in resilience and meaning‐making. Understanding these spiritual dimensions is essential for comprehending the broader context of the nursing workforce in crisis and disaster response, including nurses' motivations and resilience.

Globally, nurses' participation in disaster response is largely influenced by humanitarian values and a strong commitment to helping others during times of crisis (Hustinx et al. 2010). Previous research has also identified additional motivating factors, including personal growth, self‐esteem, professional development, the search for meaning and job satisfaction and the enhancement of interpersonal and communication skills (Powers and Daily 2010; Pring and Roco 2012). Some nurses perceive their voluntary engagement not merely as a professional responsibility but as a spiritual journey through which they experience personal growth, transcendence, job satisfaction and a deeper sense of meaning in life (Noviana et al. 2016).

In this context, spirituality is understood as an individual quest for meaning and purpose that may or may not be rooted in religion and that provides coherence, depth and hope in life (Baldacchino and Draper 2001). Whether expressed through a relationship with God, religious practice, or service to humanity, spirituality can foster resilience, give meaning to suffering and support sustained commitment (Murgia et al. 2022; Celano et al. 2022).

Disaster experiences may either challenge or reinforce individuals' spiritual beliefs. Research suggests that those with deeply internalised spiritual beliefs are better able to cope with crises, find meaning in suffering and sustain their voluntary service (Lalani et al. 2021). Consequently, spiritual beliefs function not only as a source of psychological coping but also as an intrinsic motivational force for volunteering.

From a theoretical perspective, Divine Motivation Theory (DMT) provides a philosophical foundation for understanding the source of such intrinsic motivation. According to DMT, moral values—including right actions, good outcomes and virtuous traits—are grounded in divine motives, such as love and compassion. These divine motives serve as the metaphysical foundation of ethics. Human motives are considered good insofar as they reflect divine motives within the limitations of finite and embodied beings. Thus, divine motivation in humans refers to expressions of love, compassion and justice that mirror God's motives (Zagzebski 2013).

Spirituality is a fundamental aspect of life across cultures and is shaped by religion, culture, social pluralism, history and individual perspectives (Murgia et al. 2022).

In the Iranian context, spirituality and religiosity are widely present in social and professional life and understanding their role in volunteerism is therefore particularly relevant. Accordingly, this study aimed to explore how spirituality functions as an inner driving force that motivates Iranian nurses to engage in voluntary disaster response and shapes their actions in challenging situations.

This qualitative study was guided by the following research question: How do spirituality and divine motivation inspire Iranian nurses to volunteer in disaster response and in what ways do these beliefs shape their actions and experiences in challenging situations?

## 2 Methods

### 2.1 Design

This study employed a qualitative design using conventional content analysis to describe phenomena by exploring individuals' experiences and perceptions, with particular attention to the subject under investigation (Lindgren et al. 2020). This approach enables the identification of both explicit and latent meanings within participants' narratives.

### 2.2 Data Collection and Participants

Nurses were recruited from hospitals and healthcare centres using purposive sampling. A list of nurses who had voluntarily participated in disaster situations, either domestically or internationally, was obtained. Formal inquiries were also made to the Iranian Red Crescent Society, the Nursing Services Management Office and the Deputy of Nursing at the Ministry of Health. Following the first interview, additional participants were recruited through a combination of purposive and snowball sampling, whereby initial participants referred other eligible volunteers who were willing to participate in the study.

Maximum variation sampling was used to capture diverse experiences across disaster types (e.g., floods, earthquakes, the COVID‐19 pandemic and war), living conditions, educational background and work experience. A total of 19 disaster volunteer nurses met the inclusion criteria: having at least one experience of voluntary participation in disaster response, understanding the aim of the study, willingness to provide detailed accounts of their experiences and current employment in healthcare centres affiliated with the Ministry of Health. None of the participants were members of a formal disaster response team. Two participants were excluded because they were unable to complete the interviews.

Interviews were conducted over approximately 6 months, from September 2024 to March 2025, at participants' workplaces according to their preferences, either face‐to‐face in a quiet setting or via social media platforms such as WhatsApp and Eita. Face‐to‐face interviews were conducted by both authors, whereas telephone and social media interviews were conducted by the first author under the supervision of the corresponding author.

The researchers had no prior personal relationships with the participants. The study objectives were explained clearly, and participants were informed that the study formed part of a doctoral dissertation in nursing. The interviews were conducted by two researchers: a female PhD candidate in nursing (first author) and an associate professor (corresponding author). Both researchers had extensive experience in qualitative research, content analysis, nursing education and supervision of related doctoral dissertations.

Each interview lasted between 37 and 69 min and was conducted in one or two sessions depending on participants' availability. Follow‐up interviews were conducted when necessary. All interviews were audio‐recorded with participants' consent and transcribed verbatim to ensure accuracy. Data management and analysis were conducted using MAXQDA 2020 software.

Data collection continued until data saturation was achieved. Participants were informed of their right to withdraw from the study at any time, and their data would be deleted upon request.

Demographic information was collected at the beginning of each interview. In‐depth, semi‐structured questions guided the interviews, including: ‘How have your spiritual and religious beliefs influenced your decision to volunteer and your voluntary service? Could you describe your experiences of connecting with God and the spiritual feelings you experienced while volunteering in disaster situations?’ Probing questions were used to encourage deeper reflection. At the end of each interview, participants were invited to add any further comments.

### 2.3 Data Analysis

Qualitative content analysis was conducted following Lindgren et al. (2020), in five steps:

1. Transcribing the interviews verbatim and reading the transcripts repeatedly to gain an overall understanding of the content
2. Identifying and extracting meaning units from the text
3. Condensing the meaning units and assigning codes to them
4. Grouping the codes into subcategories through constant comparison based on similarities and differences
5. Developing overarching themes to capture the latent content of the data

To ensure methodological rigour and reduce the potential for researcher bias, a systematic inter‐coder process was used. The two researchers, the first author (a PhD candidate in nursing) and the corresponding author (an associate professor), independently coded all transcripts without prior consultation. Each researcher generated initial meaning units and codes separately. They then compared their coding frameworks and resolved interpretive differences through discussion and consensus. When agreement could not be reached, a third researcher with expertise in qualitative content analysis acted as an independent arbitrator.

To further strengthen credibility and rigour, several strategies were used, including peer debriefing with the third researcher at different stages of analysis, reflective journaling by the two primary researchers to document evolving interpretations and monitor bias, and continuous comparison of interpretations with the original quotations to ensure that findings remained grounded in the data.

### 2.4 Trustworthiness

To ensure rigour and trustworthiness, Lincoln and Guba's four criteria, namely credibility, dependability, transferability and confirmability, were applied (Elo et al. 2014). Credibility was enhanced through prolonged engagement in the field and maximum variation sampling to include participants with diverse backgrounds and disaster experiences. Member checking was conducted by sharing preliminary findings with a subset of participants to verify that the interpretations accurately reflected their experiences.

Dependability was strengthened through the structured inter‐coder process described in Section 2.3, in which two researchers independently coded all data prior to comparison, resolved disagreements through discussion and consulted a third‐party arbitrator when consensus could not be reached. Themes were refined iteratively through regular team meetings, during which codes and categories were reviewed, merged, divided and renamed until a stable and coherent thematic structure was achieved.

Transferability was addressed through maximum variation sampling and the provision of rich, contextual descriptions of participants and settings, enabling readers to assess the applicability of the findings to other contexts.

Confirmability was ensured by maintaining a transparent audit trail documenting all stages of the research process, from data collection through analysis and participant feedback (Lindgren et al. 2020; Elo and Kyngäs 2008).

### 2.5 Ethical Considerations

This study was conducted in accordance with the principles of the Declaration of Helsinki and was approved by the Ethics Committee of Golestan University of Medical Sciences (Ethics Code: IR.GOUMS.REC.1403.127). Prior to data collection, the study objectives were clearly explained to the participants and the voluntary nature of participation, including the right to withdraw at any time without consequence, was emphasised. Interviews were conducted at times and locations mutually agreed upon by the researchers and participants to ensure privacy and comfort. Written informed consent was obtained from all participants, including permission to audio‐record the interviews. Strict confidentiality and anonymity were maintained throughout all stages of the study.

## 3 Findings

A total of 19 volunteer nurses participated in this study, comprising 11 men and 8 women, who had engaged in voluntary service during various disasters both within and outside Iran. The participants' mean age was 46.42 ± 9.34 years (range: 32–65 years), and their mean work experience was 19.79 years. On average, all participants had volunteered for more than 2 weeks (see Table 1).

**TABLE 1:** Demographic characteristics of the study participants.

Participant (Nurse)	Age	Gender	Marital status	Child	Total experience (years)	Education	No. of deployments	Type of disasters
1	39	Female	Married	2	6	Bachelor	1	Covid‐19
2	32	Female	Married	1	5	Bachelor	1	Earthquake
3	37	Male	Married	1	12	Bachelor	2	Covid‐19, Flood
4	47	Female	Married	2	20	Master	2	Flood
5	41	Female	Married	0	8	Bachelor	2	Covid‐19, Flood
6	41	Male	Married	1	15	Bachelor	3	Earthquake, Flood
7	43	Male	Married	1	18	Bachelor	4	Flood
8	51	Female	Married	1	20	Bachelor	2	Flood
9	49	Male	Married	3	27	Bachelor	2	Earthquake
10	46	Male	Married	2	23	Master	2	Covid‐19, Flood
11	38	Male	Single	0	13	Master	2	Covid‐19, Flood
12	46	Female	Single	0	21	Bachelor	2	Flood
13	43	Male	Married	1	23	Bachelor	4	Earthquake
14	65	Male	Married	3	26	PhD	2	War
15	62	Male	Married	2	28	Bachelor	3	War
16	67	Male	Married	0	22	PhD	2	War
17	48	Female	Married	2	21	Master	2	Covid‐19, Flood
18	36	Female	Married	2	23	PhD	1	Covid‐19
19	61	Male	Married	2	29	PhD	1	War

**FIGURE 1:**
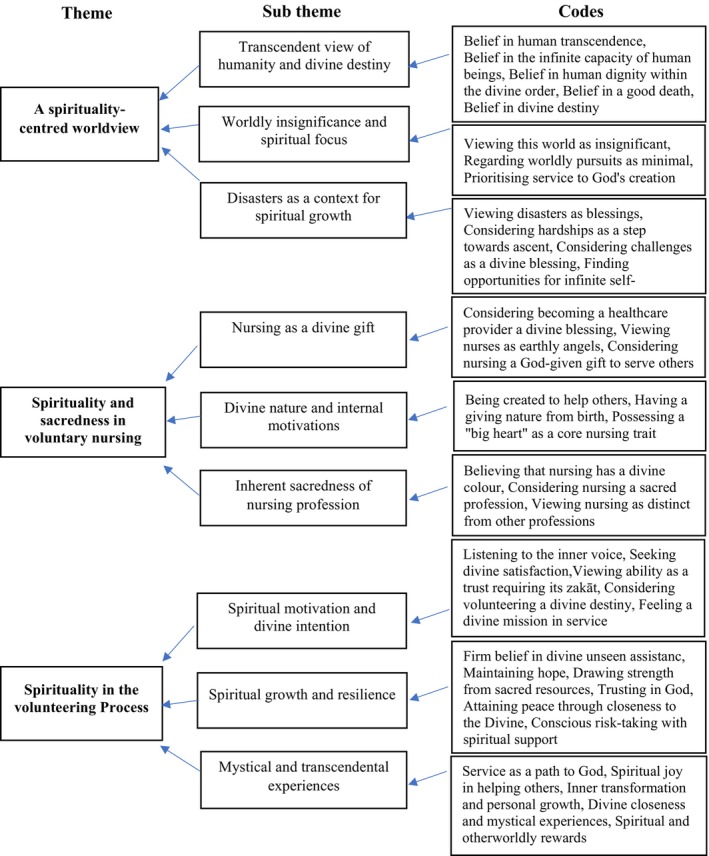
Structure of themes, subthemes and codes related to spiritual beliefs in Iranian nurses' disaster volunteering.

The findings were categorised into three main themes and nine subthemes (see Figure 1), each of which is described in detail below.

### 3.1 A Spirituality‐Centred Worldview

A spirituality‐oriented worldview emerged as one of the core concepts in nurses' understanding of the role of spirituality in motivating their voluntary participation during disasters. This theme encompassed three main categories:

#### 3.1.1 A Transcendent View of Humanity and Divine Destiny

Participants viewed humans as beings beyond mere material existence—endowed with the capacity for transcendence and infinite development, a potential that finds its fullest meaning within the framework of divine will and power. As one nurse described:

I believe that humans can be transcendent and elevated; they can experience the world, yet also go beyond it. If God created humans and endowed us with the capacity for infinity, then nothing less is truly valuable for us. This transcendent and elevated perspective, which goes beyond worldly concerns, can greatly enhance a person. (Nurse 1)

This transcendent perspective was further evident in their attitudes towards death. Participants referred to the futility of worldly attachments, viewing death as an integral part of a broader journey of growth and service. One nurse explained:

We have a duty to save lives. You have to accept it—what else would you do with the rest of your life? If I just stay and refuse, whatever is meant to happen will happen. We constantly face death… as the saying goes, ‘If we are to die, let us die as martyrs.’ (Nurse 6)

In their view, humans possess a divine essence and a transcendent outlook can elevate them beyond ordinary existence.

#### 3.1.2 Worldly Insignificance

The spiritual beliefs of Iranian nurses, rooted in their religious convictions, reflected the view that the highest purpose in life is to serve God's creation and seek closeness to the Divine—rather than becoming preoccupied with worldly possessions. As one nurse stated:

A human being is so valuable that only God is worthy of their true value. Many people devote themselves to this world—working merely for money or status—but when someone seeks infinity, they realize that the world is insignificant to them. (Nurse 1)

From their perspective, volunteering was thus understood as a divine opportunity to fulfil one's moral debt to humanity and transcend material attachments:

This opportunity allows them to partly fulfil their religious duty. When a person thinks in this way, letting go of many things may become easier for them. (Nurse 19)

#### 3.1.3 A Platform for Spiritual Growth

Volunteers viewed disasters through a spiritual lens—not as threats, but as opportunities for growth along the path of spirituality, self‐purification and closeness to God. As one nurse described:

When I look at existence with this worldview, the surrounding phenomena guide me. My perspective on disasters changes, and I see them as opportunities to achieve my goal—a meaningful path towards God. These situations deepen my sense of empathy and spirituality, and I consider them a way of spiritual growth. (Nurse 1)

Despite the hardships they entailed, disasters were perceived as opportunities for character development and enduring challenges in pursuit of spiritual elevation:

Disasters are opportunities for growth. They offer personal development… If you know where you're headed, every hardship becomes a step towards ascension. (Nurse 3)

### 3.2 Spirituality and Sacredness in Voluntary Nursing

The theme of spirituality and sacredness in voluntary nursing emerged as another central concept in the nurses' interviews, particularly within disaster contexts. This theme encompassed three main categories:

#### 3.2.1 Nursing as a Divine Gift

Participants did not perceive nursing merely as a profession; rather, they regarded it as a divine gift and a divine mission. They perceived themselves as intermediaries appointed by God to alleviate human suffering. One participant shared:

God has created each of His servants differently and has given every servant a mission. When I was granted the blessing of becoming part of the healthcare team, I saw it as a divine privilege—an opportunity to bring comfort to God's creations. I see myself as a missioned agent of God on earth… what we call angels. (Nurse 13)

#### 3.2.2 Divine Nature and Internal Motivations

Nurses' motivation to engage in voluntary service stemmed from their God‐given nature and an internalised commitment to helping others—an impulse they believed was divinely inspired, one that transcended any material reward. As one nurse described:

We were all created with a certain nature and essence from God's existence. The spirit of God has been breathed into every human being. God poured a part of His generosity into me—that's why I help this way. This act—it belongs to God Himself. It's Him. He gives permission, He wills it, He decides this path, and He chooses His servants for it. (Nurse 5)

In this view, volunteering was regarded as an inherent calling and a form of divine selection—an impulse deeply rooted and often nurtured from childhood:

It must be in the nature of the person. We have the background of these things in our family. It runs in our blood; my whole family are self‐sacrificing. (Nurse 2)

#### 3.2.3 The Inherent Sacredness of the Nursing Profession

Participants believed that voluntary nursing carried an inherent sacredness that extended beyond material rewards. For them, such service represented both an act of worship and a covenant with God:

If a disaster happens, I will volunteer with love. Payment does not matter—because in this profession, you make a deal with God. Your accountability is to God alone. (Nurse 5)

Volunteering was further understood as an expression of sincerity and spiritual devotion:

We have gained the ability, and must give its *zaka*t (obligatory almsgiving in Islam)—voluntary help done sincerely for God and others. This is stronger in nurses, perhaps because they are less influenced by materialism. (Nurse 14)

At times, volunteering also served as an expression of gratitude towards divine mercy, especially for personal protection and well‐being during disasters. One nurse shared:

The entire city got COVID, and there I was—working with sixty infected patients—and I didn't catch it. So clearly, it's You [God] who's protecting me. And I, being aware of this, must show gratitude. This is my way of giving thanks; this is what volunteering means to me. It gives you reassurance and encouragement and brings joy and a sense of fulfilment. (Nurse 10)

These narratives demonstrate that, in the minds of many nurses, voluntary disaster response was not merely a professional duty but also a spiritual commitment and a profound sacred bond with God and His creation.

### 3.3 Spirituality in the Volunteering Process

One of the core themes in the nurses' experiences was the spirituality embedded in the volunteering process, reflecting their religious beliefs and their inner connection with God throughout their service journey. For many, volunteering was not merely a professional obligation but rather a form of worship, a divine mission and a path to spiritual growth. This theme was developed through three main categories:

#### 3.3.1 Spiritual Motivation and Divine Intention

Nurses described their decision to volunteer not merely as a personal choice, but as one stemming from a deep spiritual feeling—what they interpreted as a divine calling. As one nurse shared:

It's God's will—He is the one who chooses His servants. Not everyone is granted this opportunity. When you are chosen, you should feel honoured and take pride in it. (Nurse 13)

Another nurse echoed this inner experience, describing it as ‘a voice inside me’ urging her to help (Nurse 4). From this perspective, volunteering during disasters was perceived as a divine privilege—a blessing granted to certain nurses:

For years, many have longed to serve in this way, and now God has granted us this privilege. It is not just that we desire to serve—God, too, wills to see us in service. (Nurse 19)

#### 3.3.2 Spiritual Growth and Resilience

For nurses, the experience of volunteering became a space for spiritual and personal growth. Through spiritual practices such as prayer and contemplation, nurses found that their presence in disaster settings deepened their faith, patience and psychological and physical resilience. As one nurse explained: “When you keep your eyes on that divine goal, you strive and endure the hardships. Every day I prayed, asking for physical, mental, and spiritual power.” (Nurse 1).

Faith and trust in God served as inner forces that enabled them to cope with fear and anxiety: “I surrendered myself to God, saying, ‘God, I leave myself in Your hands.’ After that, I no longer worried about my life.” (Nurse 2). Another added: “I believe that if I take one step, God will support me. I put my trust in Him, and He helps me.” (Nurse 7).

Several nurses described experiencing spiritual elevation—a sense of rising to a higher level and drawing closer to a transcendent purpose in life:

Helping beyond your formal duties feels completely different; you feel more spiritual. I felt that I had become a better person. I told myself, ‘It is good that I have truly become a good human being.’ (Nurse 12)

#### 3.3.3 Mystical and Transcendental Experiences

Participants' profound spiritual experiences during volunteering were accompanied by a sense of participation in a divine plan and an overwhelming sense of closeness to God. One nurse shared:

When you help God's creation, you become God's hand, God's colleague; it means you are getting closer to God, becoming more gentle, more compassionate. When you feel closer to God, all aspects of your spirituality progress. (Nurse 3)

Being present in those terrifying moments—when their lives were at risk—was a deeply meaningful and emotionally significant experience for the nurses:

My feeling during the war and the COVID‐19 pandemic was one of wonder…We saw nothing but beauty (*Rāʾayna illā Jamīla—*an Islamic expression denoting the perception of grace and beauty amid hardship). It truly felt like we were meant to be there. The nursing profession reveals itself more in disasters and emergencies. (Nurse 15)

Volunteering had a lasting impact on nurses' personal lives, shaped by their spiritual beliefs and cultural background, as one nurse reflected: “I volunteered during the COVID‐19 pandemic and they named me a model nurse. But I did not care about recognition. As the Persian saying goes, ‘Do good and throw it into the Tigris; God will return it to you in the desert.’ God watches over us, and I have always relied on Him.” (Nurse 11).

Nurses also pointed to personal growth and an awareness of worldly impermanence after witnessing disaster scenes:

Volunteering in disasters matured me. No matter how much wealth you have, God does not play with anyone. It was good for my personal growth. (Nurse 10)

Spirituality emerged as a key source of motivation and transcendence among volunteer nurses. Witnessing patients' calmness and faith—even across religious differences—enhanced nurses' inner peace and spiritual connection. One nurse noted:

Even though we were strangers and unfamiliar with their religion, their peacefulness enabled us to care for them with greater ease, and we felt a deep sense of spiritual connection. (Nurse 6)

Another nurse added:

The empathy and calmness of the people moved me, and it strengthened my own spiritual resolve during service. (Nurse 12)

## 4 Discussion

The findings of this study suggest that spirituality plays an important role in motivating Iranian nurses to engage in voluntary disaster response. Participants perceived volunteering not only as a professional or humanitarian duty, but also as an experience grounded in religious beliefs, divine motivation and spiritual meaning. The extracted themes point to three main elements shaping nurses' understanding of voluntary participation in disasters: a spirituality‐centred worldview, the sacredness of nursing and intrinsic experiences related to God.

Overall, many nurses perceived disaster volunteering as a form of worship, the fulfilment of a divine duty, a response to a perceived divine calling and a pathway towards spiritual growth. This interpretation appears to have been shaped by participants' religious beliefs and cultural context.

The findings presented in this study were derived from the qualitative phase of a broader instrument development study titled ‘Design and Psychometric Evaluation of an Instrument Measuring Nurses' Willingness to Participate Voluntarily in Disasters’. The present study specifically explores the role of spirituality and divine motivation among volunteer nurses.

The theme of a spirituality‐centred worldview suggests that nurses' willingness to volunteer in disasters was not viewed merely as a human response or an emotional reaction, but as a deeper expression of inner calling and spiritual conviction. This finding is consistent with studies describing spirituality as a dynamic dimension of human existence associated with meaning, transcendence and connection with self, others and God (Lalani et al. 2021; Papadopoulos et al. 2022; Baldacchino and Draper 2001; Memaryan et al. 2016). Within the Islamic context, spirituality is understood as a divine dimension of human existence that guides individuals towards closeness to God.

The pursuit of meaning and purpose in life through belief in a higher power has been emphasised in numerous studies (Noviana et al. 2016; Sloand et al. 2013; Baldacchino and Draper 2001; Papadopoulos et al. 2022; Ormsby and Harrington 2003; Memaryan et al. 2016). According to the conceptual model of spirituality, two dimensions can be identified. The vertical dimension refers to one's relationship with God or a transcendent power, which provides meaning, hope and comfort during disasters. In the present study, this dimension was reflected in nurses' trust in God's will. The horizontal dimension refers to a sense of connection and belonging to family, community, or spiritual groups (Ormsby and Harrington 2003). Here, it was expressed through commitment to professional values, social responsibility and willingness to serve during disasters.

Nurses' spiritual experiences appeared to emerge through the integration of these two dimensions, linking faith with commitment to the human community. Krok's study similarly showed that religious and spiritual frameworks can shape individuals' life goals and guide their spiritual efforts (Krok 2008). At the same time, spirituality extends beyond formal religion and may be experienced even without institutional affiliation or belief in God (van Buren et al. 2020; Gschwandtner 2021).

Individuals may take part in religious practices without strong personal religiosity, or experience spirituality in non‐religious settings (Gschwandtner 2021). Thus, religiosity does not necessarily imply deeper spirituality and the absence of religion does not preclude spiritual experience. Although this study was conducted in a religious context, the degree and expression of belief varied among participants. Individuals may participate in religious ceremonies without strong personal religiosity, or experience spirituality in non‐religious settings (Gschwandtner 2021). Thus, religiosity does not necessarily imply deeper spirituality, and the absence of religion does not preclude spiritual experience. Although this study was conducted within a religious context, participants varied in the strength and expression of their religious and spiritual beliefs.

Religion and spirituality have historically inspired responsibility and professional commitment across many societies (van Buren et al. 2020), a pattern observed particularly in cultures with collectivist values and strong religious identities (Hofstede et al. 2010). Although the present findings cannot be generalised to all nurses or to Iranian society as a whole, the volunteer nurses in this study viewed disaster service not merely as a professional duty, but also as an opportunity for spiritual growth and a profound sense of divine connection.

Their belief in the transient nature of worldly life oriented them towards the promise of spiritual reward in the hereafter. This perspective may have helped them transcend fears of death and personal hardship, thereby strengthening their motivation to engage in voluntary disaster work as a meaningful source of spiritual fulfilment.

This finding aligns with evidence suggesting that courageous behaviour is shaped by perseverance and the ability to act despite fear and perceived limitations (Mert et al. 2025). Accordingly, spiritual beliefs may have strengthened participants' perseverance, enabling them to continue engaging in disaster response despite significant risks and uncertainty.

Similarly, Ren et al. (2018) found that many rescuers confronted with sudden destruction reported a sense of meaninglessness in life. Furthermore, Naghibzadeh et al. (2025) reported that some nurses experienced religious doubts and spiritual despair. In contrast, participants in the present study perceived volunteering as a deeply meaningful practice that fostered spiritual fulfilment in a lived and tangible way. They described their role as a form of divine partnership in preserving human lives, accompanied by a strong detachment from material concerns.

For many volunteer nurses, nursing was perceived not merely as a job, but as a divine gift and an opportunity to fulfil a divine mission. They saw themselves as divinely appointed helpers whose volunteer role represented a sacred response to human suffering. This perception aligns with the concept of a religious calling, which can serve as a powerful internal motivator for altruistic behaviour (Mahmoodishan et al. 2010; Krok 2008; Baldacchino and Draper 2001). Such a sacred worldview has been interpreted as a form of intrinsic motivation embedded in nurses' professional identity, where sustained engagement in caregiving fosters an internalised disposition towards compassionate and courageous practice. Similarly, disaster‐related courageous behaviours have been conceptualised as instinct‐like responses shaped by prolonged professional exposure to patient care (Mert and Koksal 2025). However, whereas the present study interprets this internal disposition through a spiritual and transcendent lens, existing literature often explains it through professional socialisation and identity formation. Despite these differences, both perspectives point to deeply internalised behaviours driven by intrinsic rather than external motives.

Nurses who held this sacred perspective engaged in voluntary disaster response with strong intrinsic motivation, viewing their desire to help as an expression of divine calling rather than a pursuit of material incentives. Such beliefs appeared to foster responsibility, spiritual growth and resilience during disasters (Davoodvand et al. 2017). Nursing has also been portrayed as a divine mission and spiritual calling in which caring for patients reflects faith, perseverance and alignment with God's purpose (Biber 2023).

Papadopoulos et al. (2022) emphasised that disaster contexts provide a unique setting in which spirituality can function as a vital resource for healthcare providers. The present findings further suggest that participation in disaster response was experienced as a source of meaning, gratitude and spiritual fulfilment, reinforcing the sustaining role of faith in professional commitment. Consistent with these findings, Mert and Koksal (2025) reported that volunteer nurses were motivated by a desire to help others, contribute to society, fulfil a sense of responsibility and achieve spiritual satisfaction. Together, these findings suggest that disaster volunteering is driven not only by professional obligations, but also by deeper moral and spiritual values.

The sense of meaning and inner satisfaction experienced during voluntary service has been described in previous studies as a distinctive emotional state (Akbari et al. 2024; Noviana et al. 2016; Sloand et al. 2013) and as a unique emotional experience emerging from humanitarian actions (Akbari et al. 2024). Such profound emotions are often associated with a sense of connection to something beyond the self (Noviana et al. 2016).

Many nurses considered voluntary service sacred and likened it to an act of worship, describing their experiences in terms of a reciprocal spiritual commitment and a profound sense of gratitude for divine blessings. This perspective aligns with the conceptual model of spirituality (Ormsby and Harrington 2003), which highlights the role of spirituality in generating purpose, motivating service and fostering resilience. The findings suggest that spiritual beliefs served as a strong intrinsic motivator, inspiring nurses to serve with divine love and commitment. Previous studies have confirmed the role of religious beliefs, moral conscience and spiritual commitment in shaping professional behaviour among Iranian nurses (Varasteh et al. 2022; Zarei et al. 2020), while concepts such as sacred duty, religious commitment, and divine mission have been linked to endurance during disasters (Baldacchino and Draper 2001; Papadopoulos et al. 2022; Memaryan et al. 2016). Baldacchino and Draper (2001) conceptualised spirituality as a human resource that supports the pursuit of meaning, purpose, hope and inner strength. They identified four core spiritual coping strategies: connection with the self (e.g., meditation and introspection), connection with others, connection with a transcendent power or God and connection with nature. The present findings are consistent with this model, as participants reported coping strategies such as trust in God, purposeful and meaningful volunteering, a sense of mission and a moral‐humanitarian outlook towards service.

These strategies appeared to reduce psychological distress and enhanced resilience while also providing a framework for spiritual growth, inner satisfaction and life purpose. In the context of Iranian nurses' volunteering, spirituality therefore seems to be more than an individual or psychological experience; it forms part of their professional, cultural and religious identity. In a society where serving others is often viewed as a path towards divine closeness and spiritual transcendence, this perspective may be particularly significant. Strengthening the spiritual dimensions of nursing may help enhance motivation, job satisfaction and resilience during disasters.

Supporting this view, Celano et al. (2022) and Papadopoulos et al. (2022) emphasised the importance of institutional support for spirituality in nursing. At the same time, spirituality may contribute to spiritual distress under certain circumstances. During overwhelming and demanding disaster situations, the spiritual resilience of healthcare responders may weaken, particularly when clinical decisions conflict with their ethical, professional and spiritual values. In such cases, negative spiritual coping may emerge, including feelings of dissatisfaction with God, perceptions of injustice or futility and interpretations of the COVID‐19 pandemic as a form of divine retribution (Amin 2025).

The findings of this study are also consistent with the conceptual model of spirituality developed for Royal Australian Air Force nurses (Ormsby and Harrington 2003) and with the study by Papadopoulos et al. (2022), both of which highlight spirituality as a key factor influencing nurses' decision‐making, resilience and sense of professional meaning. Nurses who drew on faith and inner values attributed greater purpose and significance to their service. Similarly, a sense of divine mission and spiritual beliefs emerged as major motivators for sustained voluntary engagement during disasters.

Supporting these findings, studies in diverse disaster settings have shown that nurses often experience substantial psychological and emotional burdens, including fear, guilt, anxiety, burnout, fatigue, secondary trauma, insomnia and symptoms of post‐traumatic stress disorder, some of which may persist long after the disaster has ended (Mounsey et al. 2016; Ren et al. 2017; Akbari et al. 2024; Koksal et al. 2025). In such contexts, spirituality serves as a protective factor by promoting positive emotions and reducing physical and psychological distress (Noviana et al. 2016; Bhaskar and Mishra 2019). Participation in disaster response not only enabled nurses to fulfil professional duties but also provided opportunities to strengthen faith, achieve inner growth and enhance resilience. Spirituality was perceived as both a driving force and a source of refuge in the face of adversity.

Consistent with previous research, this study supports the dual role of spirituality for volunteer nurses in disasters—as both a source of motivation and a protective resource. Drawing on beliefs such as trust in God (*tawakkul*), divine destiny (*qadā and qadar*), faith and hope for divine reward, nurses found the strength to serve voluntarily and endure adversity (Krok 2008; de Diego‐Cordero et al. 2022; Rajabipoor Meybodi and Mohammadi 2021; Pirutinsky et al. 2021; Prazeres et al. 2021; Bhaskar and Mishra 2019).

Spiritual coping strategies—including prayer, remembrance (dhikr), meditation, consultation with religious leaders, participation in rituals, patience, moral values and reflective spiritual dialogue—provided psychological comfort, meaning‐making and a sense of spiritual connection. For many participants, disaster volunteering was perceived not merely as a professional duty, but as a sacred experience rooted in spiritual service (Achour et al. 2019; Krok 2008; Prazeres et al. 2021; Habibi Soola et al. 2022; Ibrahim et al. 2020; Hirono and Blake 2017; Bhaskar and Mishra 2019; Koksal et al. 2025).

Although this study was conducted in a context where religious beliefs were prominent among participants, the findings, together with previous cross‐cultural research, suggest that there is no single cultural or national pattern in spiritual coping strategies among nurses (Murgia et al. 2022).

Participants often perceived their work as part of a divine plan in which saving lives became a sacred act and a form of spiritual partnership with a higher purpose, thereby strengthening resilience, job satisfaction, patience, personal growth and inner contentment (Krok 2008; de Diego‐Cordero et al. 2022; Rajabipoor Meybodi and Mohammadi 2021; Salaree et al. 2021).

Nurses with strong spiritual beliefs, shaped by family and community influences, tended to use more effective coping strategies, consistent with previous findings that spirituality may foster resilience and enhances perceived control over stress (Krok 2008; Rajabipoor Meybodi and Mohammadi 2021). At the organisational level, integrating spirituality into healthcare culture—including spiritual care training, open dialogue and support from religious leaders—may reduce burnout, enhance motivation and improve team performance through empathy, psychological stability and coordination (Celano et al. 2022; Habibi Soola et al. 2022).

These findings emphasise the importance of spirituality for resilience, job satisfaction and quality of care in disaster response. Health policymakers should consider developing spiritual training programs, integrating spiritual care into nursing curricula and assessing nurses' spiritual well‐being to support voluntary engagement in disasters. Supporting strategies should address nurses' spiritual and emotional needs across all phases of disasters by providing access to religious leaders, spiritual counselling, group prayer or dhikr sessions and opportunities for individual or collective spiritual practices while respecting diverse beliefs.

Interventions such as resilience training, stress management, and group support may help sustain the volunteer workforce. Early promotion of spirituality and altruism through family, education, and media may foster a culture of volunteerism among future nurses. Future research should explore non‐religious and cross‐cultural contexts to deepen understanding of the influence of spirituality on motivation and resilience across diverse settings.

## 5 Conclusion

The findings of this study suggest that spirituality, reflected in a spirituality‐centred worldview, the perceived sacredness of nursing and a sense of divine connection, was a major source of motivation for Iranian nurses' participation in disaster volunteering. From the participants, this form of service was not only a professional duty, but also a divine calling associated with self‐transcendence, closeness to God and job satisfaction.

These findings suggest that spirituality may function as both a motivating force and a protective resource against psychological stress, burnout and anxiety in disaster settings. In this cultural and religious context, voluntary service was understood as obedience to divine command, a form of charitable giving and a contribution to saving human lives.

Within the religious and cultural context described by participants in this study, voluntary service to others is imbued with meanings such as obedience to divine command, offering one's capabilities as charity (*zakat‐e tavanaei*, a form of Islamic charitable giving) and participation in saving human lives. These meanings strengthen nurses' resilience during disasters. Accordingly, spirituality is emphasised not merely as an individual trait, but as a psychological, ethical and professional foundation for sustaining the nursing workforce under disaster conditions.

### 5.1 Limitations

This study was limited to Iranian volunteer nurses, and our access to representatives of all religious minority groups was limited. As a result, variations in participants' degrees of religiosity and spiritual orientation, as well as the perspectives of nurses from minority religious backgrounds, may limit the transferability of the findings to other cultural or religious contexts. Because qualitative content analysis is inherently interpretive, the possibility of researcher bias cannot be entirely eliminated despite the rigorous measures used to minimise it. Although broader geopolitical tensions in the Middle East may have influenced participants' humanitarian motivations, this issue was not explored during the interviews and is therefore unlikely to have directly shaped the findings. Future research would benefit from mixed‐methods designs and more culturally and religiously diverse samples to deepen understanding of the role of spirituality in disaster volunteerism.

## Author Contributions

All authors contributed to the conception and design of the study. Data collection, preparation of materials, and analysis were carried out by Zahra Talebi, Gholamreza Mahmoodi‐Shan and Abbas Ebadi. Zahra Talebi prepared the initial draft of the manuscript, and all authors contributed to reviewing, editing, and refining subsequent versions. All authors read and approved the final manuscript.

## Funding

The authors have nothing to report.

## Ethics Statement

The research proposal with code 114061 was approved by the Deputy of Research and Technology at Golestan University of Medical Sciences.

## Consent

All participants provided written informed consent, and data were anonymised to protect privacy.

## Conflicts of Interest

The authors declare no conflicts of interest.

## Data Availability

The data that support the findings of this study are available on request from the corresponding author. The data are not publicly available due to privacy or ethical restrictions.
